# Twelve-Month Cognitive and Functional Outcomes Following Cardiac Surgery: The DEXACET Trial of Intravenous Acetaminophen Versus Placebo

**DOI:** 10.3389/fphar.2022.803903

**Published:** 2022-03-22

**Authors:** Tanvi Khera, Jordan Helfand, Lauren Kelly, Ariel Mueller, Puja Shankar, Edward R. Marcantonio, Balachundhar Subramaniam

**Affiliations:** ^1^ Center for Anesthesia Research Excellence, Department of Anesthesia, Critical Care and Pain Medicine, Beth Israel Deaconess Medical Center, Boston, MA, United States; ^2^ Department of Anesthesia, Critical Care and Pain Medicine, Massachusetts General Hospital, Harvard Medical School, Boston, MA, United States; ^3^ Department of Medicine, Divisions of General Medicine and Gerontology, Beth Israel Deaconess Medical Center, Harvard Medical School, Boston, MA, United States

**Keywords:** cognition, delirium, functional status, cognitive dysfunction, cardiac surgery, dexacet trial

## Abstract

**Background:** Delirium, an acute decline in attention and global cognitive dysfunction, occurs frequently following cardiac surgery and has been demonstrated to be significantly associated with cognitive dysfunction and reduced functional ability. In the DEXACET trial, we demonstrated a significant reduction in postoperative in-hospital delirium with intravenous (IV) acetaminophen when compared with placebo. In this analysis we examined whether this protective association also extended to 12 month cognitive and functional outcomes.

**Methods:** This study was a prospective, randomized, placebo-controlled, triple-blinded, factorial design trial conducted at Beth Israel Deaconess Medical Center, approved by the IRB. In this trial, 120 older cardiac surgical patients were randomly assigned to receive either intravenous (IV) acetaminophen or placebo in addition to propofol or dexmedetomidine. Those receiving IV acetaminophen displayed a significant reduction in in-hospital delirium. We collected cognitive, mood and functional outcome data using the Montreal Cognitive Assessment, telephone version (T-MoCA), Geriatric Depression Scale (GDS) and the Basic and Instrumental Activities of Daily Living (ADLs, IADLs) at 1 month and 12 months after surgery.

**Results:** Of the 120 enrolled patients in the primary trial, 93 (77.5%) and 83 (69.2%) patients responded to assessments at 1 month and 12 months, respectively. No statistically significant differences in median T-MoCA scores were observed between acetaminophen and placebo groups at 1 month (18.0 vs.18.0, *p* = 0.52) or 12 months (19.0 vs.18.0, *p* = 0.62) following surgery. There were similarly no differences in GDS, ADLs or IADLs between treatment groups. Losses to follow-up limited the sample sizes and 10 of the 23 (45%) original study participants who had postoperative delirium were lost to follow up.

**Conclusion:** Administration of intravenous acetaminophen was not associated with a difference in long term cognitive or functional status following cardiac surgery. Additional research on long-term outcomes following postoperative delirium with a larger sample size and improved cohort retention strategies will be needed to address this important area.

## Introduction

Delirium, characterized as an acute decline in attention and global cognitive dysfunction, occurs in up to 50% of patients following cardiac surgery (Rudolph and Marcantonio, 2011). It is associated with increased morbidity and mortality, prolonged length of hospital stay, postoperative cognitive dysfunction [POCD], and decline in functional status (Rudolph and Marcantonio, 2011). POCD, which is defined as a clinically significant decline in cognitive function following recovery from surgery, is a serious concern for those undergoing any form of surgery but is especially prevalent among cardiac surgery patients (Hovens et al., 2016). While the prevalence of POCD in those undergoing non-cardiac surgery is approximately 12%, the reported incidence of POCD among those undergoing cardiac surgery ranges from 20–50% at 3 months postoperatively (Hovens et al., 2016; Evered and Silbert, 2018). Recent studies have highlighted the increased risk for an accelerated course of cognitive decline following delirium at 12 and 36 months postoperatively (Saczynski et al., 2012; Inouye et al., 2016).

While the mechanisms for both delirium and POCD following cardiac surgery are likely multifactorial, well-documented risk factors include baseline cognitive impairment, psychoactive medications, pain, and inflammation related to surgery (Brown, 2014; Inouye et al., 2014; Indja et al., 2017). Although the pathophysiological mechanism of delirium is still largely unknown, it is believed that cholinergic deficiency is the final pathway. In fact, a heightened systemic inflammatory response to surgery or postoperative infection may lead to increased permeability of the blood-brain barrier with resultant neuro-inflammation, triggering both delirium and POCD. This direct injury to the neurons can lead to long term downstream adverse effects (Marcantonio, 2012). Acetaminophen has analgesic, antipyretic, antioxidant and anti-inflammatory action. Neuronal cell survival is improved in oxidatively challenged cell cultures exposed to acetaminophen (Tripathy and Grammas, 2009). Current literature comparing the formulations of acetaminophen show that IV acetaminophen offers higher CSF concentration, greater area under the curve (AUC), shorter time to reach maximum plasma concentration, improved analgesic coverage and decreased requirement for narcotic agents when used as a part of multimodal analgesia in the postoperative period (Singla et al., 2012; Politi et al., 2017). Because of its analgesic properties, as well as possible protection against neuro-inflammation, intravenous (IV) acetaminophen was considered as a potential protective factor against postoperative delirium, as well as associated longer-term sequelae (Subramaniam et al., 2019).

In the recent DEXACET trial, we demonstrated a significant reduction in delirium in older cardiac surgery patients treated with IV acetaminophen, combined with IV propofol or dexmedetomidine, compared with placebo (Subramaniam et al., 2019). We did not find differences in the rates of delirium in the IV propofol vs. dexmedetomidine arms, therefore the current analysis focuses only on the effects of IV acetaminophen. Using 12-month follow up data from that trial, this analysis investigates the relationship between IV acetaminophen and longer-term functioning following cardiac surgery. Given the significant reduction in delirium observed in the primary analysis, we hypothesized that in-hospital use of IV acetaminophen six-hourly for eight doses after cardiac surgery is associated with an improved cognitive functioning and functional status at 1 month and 12 months following surgery as compared to placebo.

## Methods

The DEXACET trial was a prospective, randomized, placebo-controlled, triple-blinded, factorial design trial, carried out from September 2015 through April 2018, with long-term follow up completed in April 2019. The purpose of the study was to assess the impact of postoperative scheduled acetaminophen and sedatives (propofol or dexmedetomidine) to decrease the incidence of postoperative delirium in elderly cardiac surgical patients. Long term follow up was an apriori aim for this study as well. This study was approved by the Committee on Clinical Investigations Institutional Review Board (Protocol 2014P000413) at Beth Israel Deaconess Medical Center (BIDMC) and was registered at clinicaltrials.gov (Protocol NCT02546765).

Full details of the study protocol and results were published previously (Shankar et al., 2018; Subramaniam et al., 2019). In brief, 120 patients age 60 years and older undergoing either coronary artery bypass graft (CABG) or CABG plus valve replacement surgery were included. Patients were excluded if they were undergoing either an emergent procedure or isolated aortic surgery, had preoperative ejection fraction less than 30%, preexisting cognitive impairment, Alzheimer’s disease, Parkinson’s disease, medications for cognitive decline, history of recent seizures, serum creatinine levels above 2 mg/dl, liver dysfunction, recent history of alcohol misuse, English language limitations, or hypersensitivity to any study medication. After confirming subject eligibility, written informed consent was obtained by a study physician. Using computer generated block randomization, patients were assigned into groups (1:1:1:1) to receive either acetaminophen and propofol, acetaminophen and dexmedetomidine, placebo and propofol, or placebo and dexmedetomidine. The dexmedetomidine group received an IV bolus dose of 0.5–1 μg/kg during chest closure, followed by a maintenance infusion of 0.1–1.4 μg/kg per hour. The IV propofol group received a maintenance dose of 20–100 μg/kg per minute. Eight Doses of acetaminophen (1 g IV) or placebo (0.9% normal saline) were administered upon immediate post-operative admission to the intensive care unit (ICU) and, repeated every six-hourly for the first 48 h after surgery. Blinding could only be achieved between the analgesic treatment arms (acetaminophen vs. placebo), not the sedation arms (propofol and dexmedetomidine).

Preoperative baseline and discharge cognitive assessments were performed in all subjects using the Montreal Cognitive Assessment (MoCA) (Nasreddine et al., 2005). MoCA consists of a 30-point questionnaire used to test the various domains of cognitive function. A briefer cognitive assessment was administered daily in the hospital to assess for delirium. This assessment included questions evaluating language, attention, concentration, working memory, short term memory, visuo-spatial abilities, abstract reasoning and orientation to time place and person (Nasreddine et al., 2005). Additionally, the Delirium Symptom Interview (DSI) was used to elicit specific symptoms of delirium, such as perceptual disturbances and delusions (Albert et al., 1992). These daily assessments in the hospital were used to score the Confusion Assessment Method (CAM), a standardized approach to identify delirium, with high sensitivity (94–100%) and high specificity (90–95%) (Inouye et al., 1990; Inouye et al., 2016). All study assessors were blinded to treatment group.

Following discharge, patients received follow-up assessments to measure cognition, mood, and functional status, predefined secondary outcomes, at 1 month and 12 months following surgery. Cognitive function was assessed using the Telephone Montreal Cognitive Assessment (T-MoCA), a 22-point validated telephone-based cognitive assessment tool that can be used remotely to follow-up patients after discharge. The T-MoCA contains a subset of MoCA items (22 out of 30) that can be administered by phone, facilitating comparison with baseline MoCA scores, from which the T-MoCA items were used for comparison. Sustained attention tasks, days of week backwards (DOWB) and months of year backwards (MOYB), were added to the T-MOCA to increase sensitivity for detection of inattention, a feature of delirium (Zietemann et al., 2017). Patients were also assessed with the short-form Geriatric Depression Scale (GDS), scored 0-15 (15 = worst); scores ≥5 indicated depressive symptomatology (Yesavage and Sheikh, 1986; Albert et al., 1992).

Functional status was evaluated during the same interview using the activities of daily living (ADL) and instrumental activities daily living (IADL) scales. The ADL scale consists of a set of questions assessing the patient’s requirement for assistance to carry out six basic daily activities: feeding, walking, toileting, bathing, dressing and grooming (Katz et al., 1970). the ADL scale was scored 0-6 (6 = independent), with a score <6 indicating any ADL impairment. IADL, an instrument that assesses higher order activities needed to maintain independent living in the community, cooking, housework, finances, medications, transportation, and telephone, was administered using a separate scale (Marcantonio, 2012); the IADL scale was scored 0-6 (6 = independent), with a score <6 indicating any IADL impairment. Declines in cognitive and functional status from preoperative baseline to follow-up time points were evaluated since they may reflect important changes in quality of life for the participants.

Following the standard practice in many epidemiologic studies to limit losses to follow up, the protocol permitted a time window for completion of the follow-up interviews of 14 days before and after the 1-month scheduled time-point, as well as 3 months before and after the 12-month scheduled time-point.

### Statistical Analysis

In this analysis, descriptive statistics of the data are presented based on variable type and distribution. Continuous data was presented as median (interquartile range [IQR]) after confirming with the Shapiro-Wilk test that data did not follow a normal distribution. Differences between groups were assessed with a Wilcoxon Rank-Sum test or Kruskal–Wallis test (for comparison of more than two groups). Categorical data are presented as frequencies and proportions and differences between groups were assessed with a chi-square or Fisher’s Exact test, as appropriate. No imputation was performed for missing data. Our primary analysis of this data focused on the association between analgesic groups (acetaminophen vs placebo). Generalized estimating equations (GEE) were performed to test whether analgesic group, time, and their interaction predicted T-MoCA, GDS, ADL, and IADL scores. An exchangeable correlation structure was used to account for repeated measures within individuals. In post-hoc analyses, we also assessed differences in cognitive and functional status between those who did and did not develop postoperative delirium during their hospital stay. Two-sided *p*-values <0.05 were considered statistically significant. SAS Version 9.4 (SAS Institute Inc, Cary, NC) was utilized for all analyses.

## Results

### Patient Characteristics by Treatment Group

Table 1 shows the baseline demographic and surgery characteristics stratified by treatment group. The baseline demographics including age, sex, ethnicity, surgical characteristics, and lengths of hospital stay were comparable between the treatment groups. The median age was 69 years (IQR 63-74) in the acetaminophen group and 70.5 years (IQR 63-76.5) in the placebo group. 83% of the participants in the acetaminophen groups were men compared to 85% in the placebo group. At the time of hospital discharge, patients in the placebo group had a significantly higher rate of delirium as compared to the acetaminophen group (10% in the acetaminophen group vs. 28.3% in the placebo group, *p* = 0.011). The median hospital length of stay for the acetaminophen group was 8 days (IQR 6-9.5) compared to 8.5 days (IQR 6-11) in the placebo group.

**TABLE 1:** Patient characteristics by treatment group. Patient characteristics are compared between treatment groups and no significant differences are found. Outcomes of delirium rate and median hospital length of stay are also compared, and a significant difference is found in delirium rate with 10% in the acetaminophen group and 28.3% in the placebo. Wilcoxon Rank-Sum tests and chi-square or fisher exact tests were utilized to compare the characteristics of the groups. (*IQR: Interquartile range, CABG: Coronary artery bypass grafting, AVR: Aortic valve replacement, MVR: Mitrial valve replacement*)

Characteristic	Acetaminophen .group (*n* = 60)	Placebo group (*n* = 60)	*p*-value
Age, median (IQR)	69 (63, 74)	70.5 (63, 76.5)	0.566
Male sex, n (%)	50 (83.3)	51 (85.0)	0.803
Self-reported race, n (%) all categories			1.0
*American Indian or Alaska Native*	0 (0.0)	0 (0.0)	
*Asian*	1 (1.7)	0 (0.0)	
*Black or African American*	3 (5.0)	2 (3.3)	
*Native Hawaiian or Other Pacific Islander*	0 (0.0)	0 (0.0)	
*White*	55 (91.7)	56 (93.3)	
*Other*	0 (0.0)	1 (1.7)	
*Multi-Racial*	0 (0.0)	1 (1.7)	
*Unknown*	1 (1.7)	0 (0.0)	
Surgical type, n (%)—all categories			0.309
*CABG*	38 (63.3)	41 (68.3)	
*CABG + MVR*	2 (3.3)	0 (0.0)	
*CABG + AVR*	18 (30.0)	14 (23.3)	
*Other*	2 (3.3)	5 (8.3)	
Delirium rate, n (%)	6 (10.0)	17 (28.3)	0.011
Number of days with delirium			
Median (IQR)	0 (0, 0)	0 (0, 1)	0.008
Mean (SD)	0.1 (0.3)	0.7 (1.3)	
Hospital length of stay, median (IQR)	8 (6, 9.5)	8.5 (6, 11)	0.126

### Comparison of Baseline Cognition by Follow up Status

**FIGURE 1:**
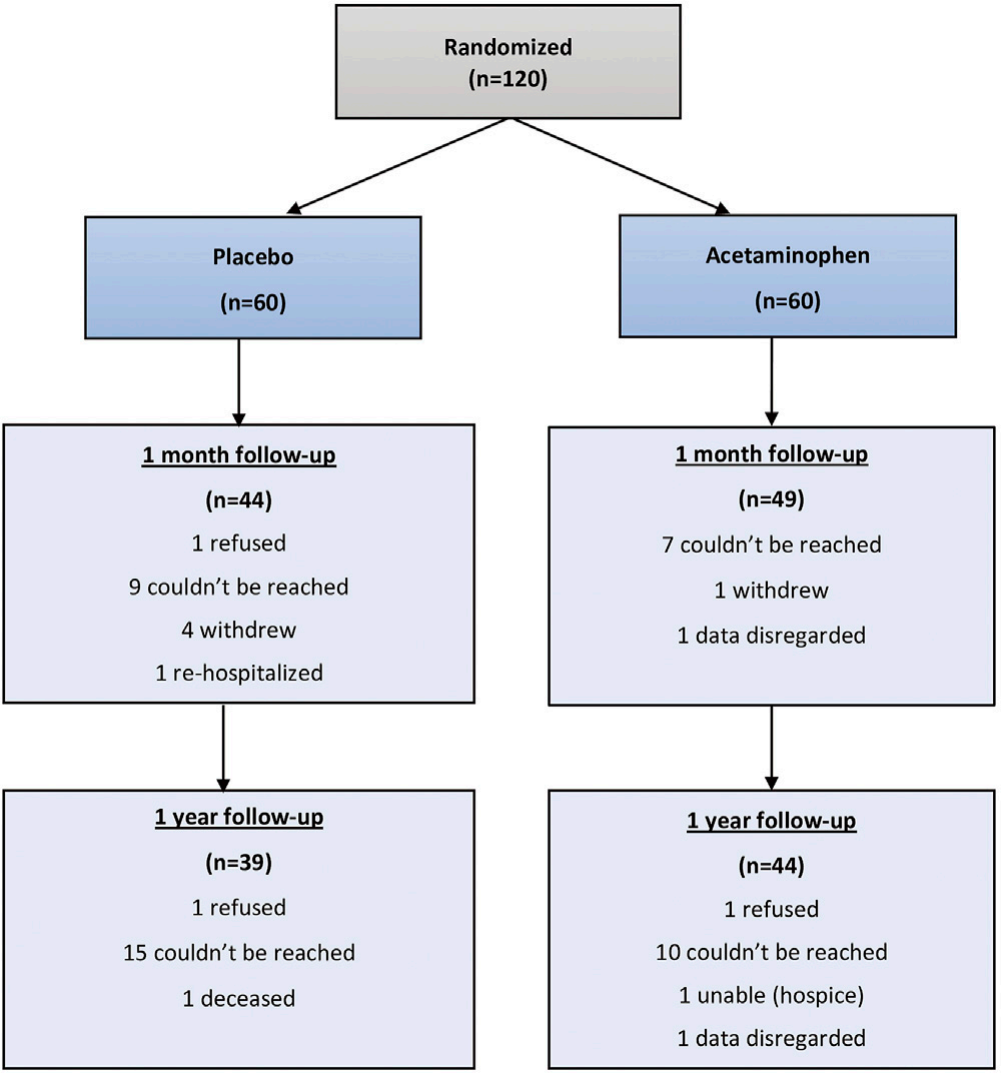
Consort diagram.

Of 120 randomized study participants, 93 (77.5%) were reached at 1 month and 83 (69.2%) were reached at 12 months. (Figure 1). At 1 month 28 patients were lost to follow-up and 92 were able to complete the T-MoCA. Median baseline MoCA scores for those who were not followed up was 17 (IQR 14.3, 18.5) and for those who were followed up was 17.2 (Yesavage and Sheikh, 1986; Albert et al., 1992), *p* = 0.1943. Similarly, at 1 year 41 patients were lost to follow up and 79 completed the T-MoCA scores. Median baseline MoCA score for those patients who were not followed up was 17.8 (Yesavage and Sheikh, 1986; Nasreddine et al., 2005) and for those who were followed up was 17 (Yesavage and Sheikh, 1986; Nasreddine et al., 2005), *p* = 0.732.

### Overall Outcomes in Entire Cohort

Within the entire cohort (N = 120), we observed a statistically significant increase in median T-MoCA scores from baseline (17.0 [IQR 15.0–19.0]) to 1 month (18.0 [IQR 16.0–20.0]) (*p* = 0.0001). At 12 months, the median T-MoCA score of 19.0 (IQR 17.0–20.0) was also significantly higher than baseline (*p* < 0.0001). Among the entire cohort, we observed a statistically significant increase in the proportion of patients with any ADL impairments at 1 month (24.7%) compared to baseline (3.3%) (*p* < 0.0001). Similarly, there was a statistically significant increase in the proportion of patients with any IADL impairments at 1 month (54.8%) relative to baseline (8.3%) (*p* < 0.0001). However, by 12 months, there was no longer a significant difference in proportion of patients with any ADL or IADL impairments (12.1 and 16.9%, respectively) relative to baseline (*p* = 0.065 and *p* = 0.144, respectively).

### Cognitive Outcomes by Treatment Group

**FIGURE 2:**
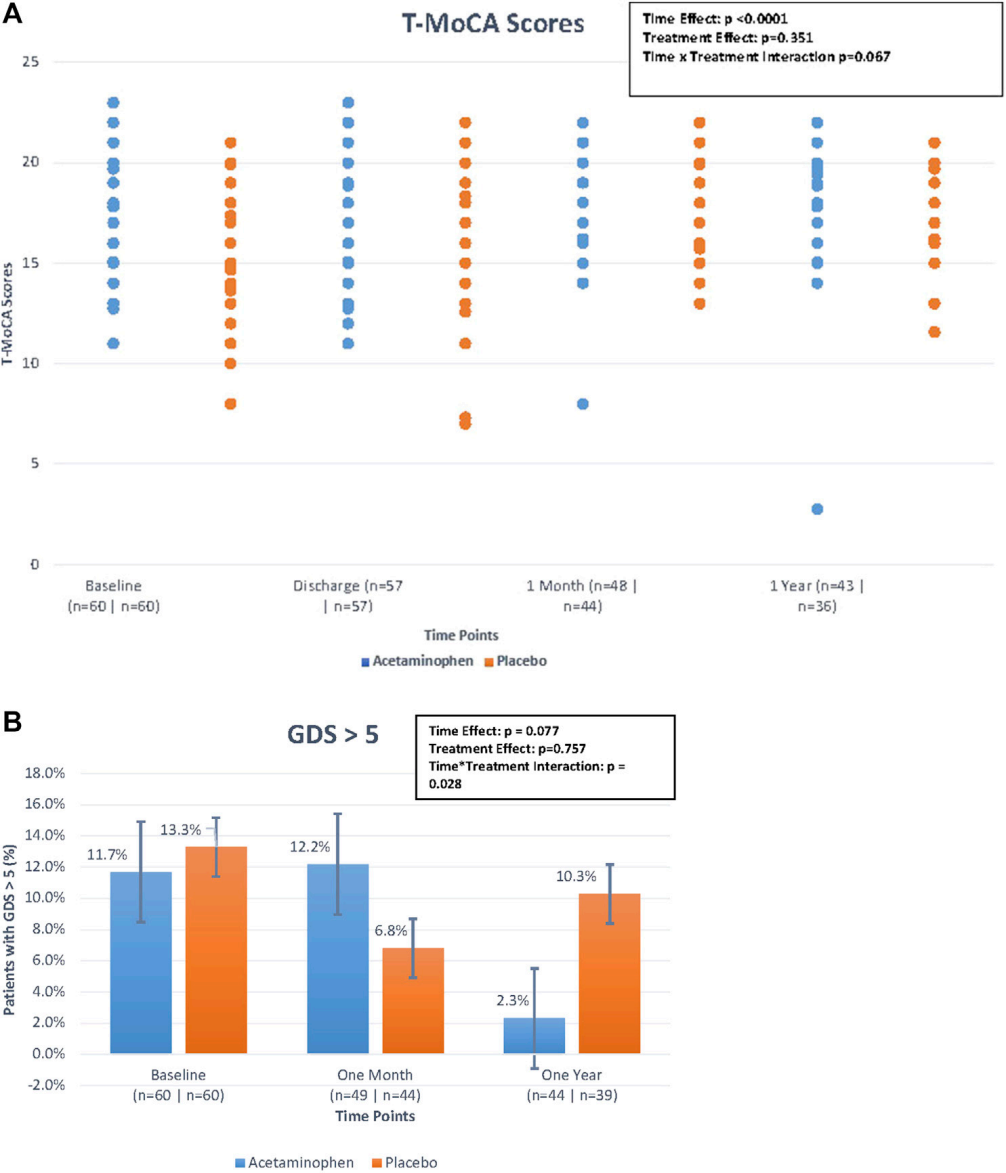
**(A)**. Median Telephonic- Montreal Cognitive Assessment (T-MoCA) scores over time by treatment group. The figure displays the differences in median T-MoCA scores between treatment groups (treatment effect), the differences in T-MoCA scores over the four time points (time effect), and the interaction of these two effects (time*treatment interaction) looking at the figure as a whole. The time effect was found to be statistically significant but the others were not. Generalized estimating equations were performed to obtain these *p*-values. **(B)** Percentage of patients with geriatric depression scale > 5 over time by treatment group. The figure displays the differences in the percentage of patients with GDS scores >5 between treatment groups (treatment effect), the differences in the percentage of patients with GDS scores >5 over the three time points (time effect), and the interaction of these two effects (time*treatment interaction) looking at the figure as a whole. Although the treatment*time interaction *p*-value is < 0.05, because the primary comparisons were insignificant, the interaction was considered insignificant. Generalized estimating equations were performed to obtain these *p*-values.

We compared T-MoCA scores over time between acetaminophen and placebo groups. At baseline and hospital discharge, median T-MoCA scores for patients in the acetaminophen group were approximately one point higher than T-MoCA scores within the placebo group. At 1 month, both groups had a median value of 18.0. At 12 months, the median T-MOCA score for the acetaminophen group was one point higher than the placebo group, a difference that did not achieve statistical significance (*p* = 0.62) (Table 2; Figure 2A). A significant time effect was demonstrated (*p* <0 .001) with increasing T-MoCA over time, likely due to learning effects; however, no significant treatment effect (RR 1.05, *p* = 0.35) or time × treatment interaction was demonstrated.

**TABLE 2:** Overall Results for Cognitive and Functional Outcomes Over Time. Generalized estimating equations were performed to test whether analgesic group, time, and their interaction predicted T-MoCA, GDS, ADL, and IADL scores. The number of patients assessed is displayed for each outcome as it varies over time and by treatment group due to withdrawals, assessments refused, etc. Median (IQR) are displayed for T-MoCA scores and Frequency (%) are displayed for GDS, ADL, and IADL scores which have been dichotomized. Risk ratios are displayed for the time effect with baseline as the reference, for the treatment effect with placebo as the reference, and for the time*treatment interaction with baseline-placebo as the reference. A significant time effect was found for T-MoCA, ADL, and IADL. Although the GDS treatment*time interaction *p*-value is < 0.05, because the primary comparisons were insignificant, the interaction was considered insignificant**.** (*T-MoCA: Telephonic- Montreal cognitive assessment, GDS: Geriatric depression scale, ADL: Activities of daily living, IADL: Instrumental activities of daily living, IQR: Interquartile range*).

Scores	Baseline	Discharge	One month	12 Months	*p*-value
***T-MoCA Score***					
*Number Assessed*	60	57	48	43	
*Acetaminophen, Median (IQR)*	17.9 (16.0, 18.5)	18.0 (16.0, 20.0)	18.0 (16.0, 20.0)	19.0 (17.0, 20.0)	
*Number Assessed*	60	57	44	36	
*Placebo, Median (IQR)*	17.0 (14.3, 19.0)	17.0 (15.0, 19.0)	18.0 (16.5, 20.0)	18.0 (16.0, 20.0)	
*Time Effect*	Ref	RR = 1.0	RR = 1.1	RR = 1.1	<0.0001**
*Treatment Effect*	RR = 1.05*	0.351			
*Treatment X Time Effect*	Ref	RR = 1.0	RR = 0.94	RR-0.96	0.067
***GDS*** ( ***> 5*** )					
*Number Assessed*	60	—	49	44	
*Acetaminophen, No. (%)*	7 (11.7)	—	6 (12.2)	1 (2.3)	
*Number Assessed*	60	—	44	39	
*Placebo, No. (%)*	8 (13.3)	—	3 (6.8)	4 (10.3)	
*Time Effect*	Ref	—	RR = 0.37	RR = 0.68	0.077
*Treatment Effect*	RR = 0.86*	0.757			
*Treatment X Time Effect*	Ref	—	RR = 3.34	RR = 0.26	0.028
***ADL Score*** ( ***< 6*** )					
*Number Assessed*	60	—	49	44	
*Acetaminophen, No. (%)*	3 (5.0)	—	14 (28.6)	6 (13.6)	
*Number Assessed*	60	—	44	39	
*Placebo, No. (%)*	1 (1.7)	—	9 (20.5)	4 (10.3)	
*Time Effect*	Ref	—	RR = 15.04	RR = 6.47	<0.0001**
*Treatment Effect*	RR = 3.11*	0.205			
*Treatment X Time Effect*	Ref	—	RR = 0.5	RR = 0.46	0.804
***IADL Score < 6***					
*Number Assessed*	60	—	49	44	
*Acetaminophen, No. (%)*	6 (10.0)	—	25 (51.0)	8 (18.2)	
*Number Assessed*	60	—	44	39	
*Placebo, No. (%)*	4 (6.7)	—	26 (59.1)	6 (15.4)	
*Time Effect*	Ref	—	RR = 19.87	RR = 2.58	<0.0001**
*Treatment Effect*	RR = 1.56*	0.755			
*Treatment X Time Effect*	Ref	—	RR = 0.47	RR = 0.77	0.561

*Reference for treatments is placebo group.

**Significant at *p* < 0.004; Applying the Bonferroni correction to all the 12 tests presented in Table 2, as part of a sensitivity analysis, alpha becomes 0.004.

### Depression Outcomes by Treatment Group

We used a standard cut-point of GDS ≥5 to compare rates of depressive symptoms across treatment groups over time. For the acetaminophen group, the rates of GDS ≥5 were 11.7% at baseline; 12.2% at 1 month, and 2.3% at 12 months; for the placebo group the rates were 13.3, 6.8, and 10.3%, respectively (Table 2; Figure 2B). While the rate of significant depressive symptoms generally went down over time, this was not significant. There was no significant treatment effect or treatment × time interaction for this analysis (please note if the primary comparisons were insignificant, the interaction was considered insignificant even if the *p*-values were <0.05).

### Functional Outcomes by Treatment Group

**FIGURE 3:**
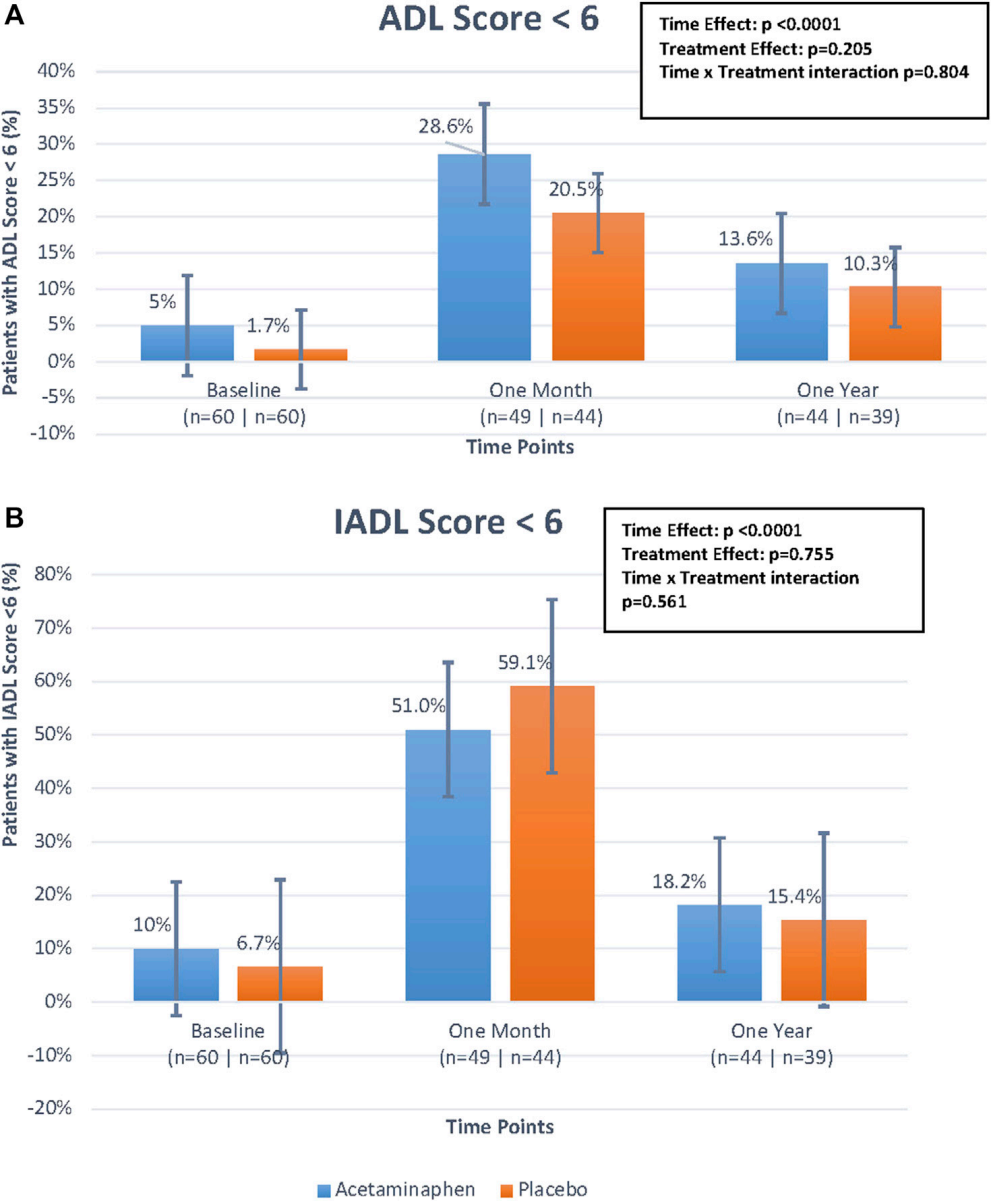
**(A)**. Percentage of patients with Activities of Daily Living (ADL) score <6 over time by treatment group. The figure displays the differences in percentage of patients with ADL scores <6 between treatment groups (treatment effect), the differences in percentage of patients with ADL scores <6 over the three time points (time effect), and the interaction of these two effects (time*treatment interaction) looking at the figure as a whole. The time effect was found to be statistically significant but the others were not. Generalized estimating equations were performed to obtain these *p*-values. **(B)**. Percentage of patients with Instrumental Activities of Daily Living (IADL) score <6 over time by treatment group. The figure displays the differences in percentage of patients with IADL scores <6 between treatment groups (treatment effect), the differences in percentage of patients with IADL scores <6 over the three time points (time effect), and the interaction of these two effects (time*treatment interaction) looking at the figure as a whole. The time effect was found to be statistically significant but the others were not. Generalized estimating equations were performed to obtain these *p*-values.

For ADL and IADL scales (0-6), we examined any functional impairment at each time-point, using a cut-point of <6 by treatment group. As already described above, both groups had a substantial functional decline between baseline and 1 month, with gradual functional improvement at 12 months (Table 2; Figures 3A,B). While the time trend was statistically significant for both ADL and IADL (*p* <0 .0001), there was no significant treatment effect in either group (*p* >0 .2) and no time × treatment interaction. Thus, treatment did not show significant effects on functional outcomes at one or 12 months in this study.

### Cognitive Outcomes by Delirium Status

**FIGURE 4:**
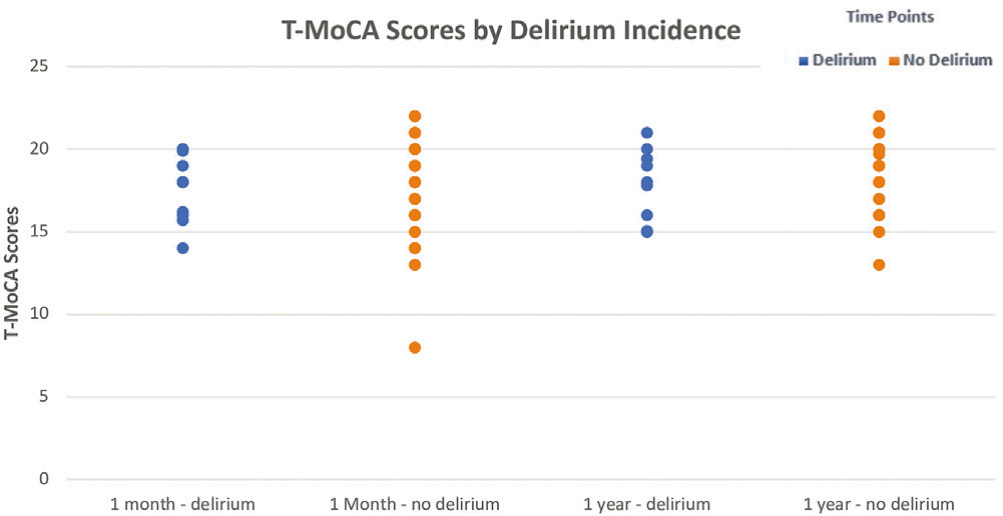
Median ± Interquartile range (IQR) Telephonic- Montreal Cognitive Assessment (T-MoCA) scores by incidence of delirium at 1 month and at 1 year The figure displays the differences in median T-MoCA scores between those with delirium and those without delirium at the two follow-up time points. While no significant differences were observed, median T-MoCA scores were slightly higher in the non-delirium group compared to the delirium group at both time points. A Wilcoxon Rank-Sum test was utilized to test this.

After 1 month, the no-delirium group had a median T-MoCA score of 18.0 (IQR 16.0–20.0) as compared to the delirium group which had a median score of 18.0 (IQR 16.2–19.0; *p* = 0.43). However, after a year the median T-MoCA score of the no-delirium group was 19.0 (IQR 17.0–20.0) while the median T-MoCA score of the delirium group was 17.9 (IQR 15.0–19.4). While no significant difference was observed, we did see an upward trend in median T-MoCA score in the non-delirium group relative to the delirium group between 1 month and 12 months (Figure 4).

## Discussion

In this analysis of the 1-month and 12-month outcomes of our clinical trial, no significant differences between acetaminophen and placebo groups were observed in cognitive or physical functioning at either time point for T-MoCA, GDS scores, ADLs, or IADLs. While we observed significant improvements between median T-MoCA scores at baseline as compared to 12 months follow-up, this could largely be attributable to the learning effects commonly associated with repeated cognitive testing (Heilbronner et al., 2010). For all measures, except GDS we observed significant time effects at 1 and 12 months relative to baseline, but there were no significant treatment effects, or time × treatment interaction effects, for cognitive, mood or functional outcomes. Although in our initial study we observed a significant association between treatment with acetaminophen and a lower rate of in-hospital delirium following cardiac surgery, this did not translate into improved cognitive or functional outcomes at 1-month or 12-months follow-up (Marcantonio, 2012).

Previous studies have documented increased rates of depression following cardiac surgery (Pourafkari et al., 2016). Importantly, both baseline and postoperative depression are strongly associated with adverse long-term outcomes and mortality following cardiac surgery (Takagi et al., 2017; Drudi et al., 2018). While we did not find any significant trends within our own cohort, it is important to continue to monitor postoperative depression closely in similar studies, particularly among the elderly.

Experimental animal models have shown improved cognition when administered acetaminophen after orthopedic surgery or post LPS–induced cognitive impairment potentially due to its central modulatory effects (Zhao et al., 2017; Garrone et al., 2021). This was demonstrated by alteration in hippocampal cytokines levels and markers of microtube dynamics. The literature for long term impact of acetaminophen, on cognition, in both experimental animals and humans, remains limited. Our study did not demonstrate a significant difference in cognitive function between the groups at 1 month and 12 months. This may in part be because of the losses to follow up that were incurred at 1 month and 12 months. Our findings regarding cognitive outcomes are similar to a prior small cohort study by Neufeld et al. examining the long-term effects of delirium following surgery with general anesthesia (Neufeld et al., 2015). In that study, no significant differences between those with and without postoperative delirium were found in cognitive ability or functional status at 18 months postoperatively (Neufeld et al., 2015). While 45% of their study population (41 people) had diagnosed delirium in the post anesthesia care unit and 96% of survivors followed-up, they did not find any significant long-term differences (Neufeld et al., 2015). However, a number of other studies have demonstrated a significant decline in cognitive ability up to 3 months following postoperative delirium, which resolved by 12 months postoperatively (Brown et al., 2018; Austin et al., 2019). We did not perform any assessments between the 1 month and 12 months period, and thus, we are unable to comment on whether a nadir occurred for these patients between our scheduled follow up assessments.

While significant declines in cognitive ability and functional ability between one and 3 months following postoperative delirium have been frequently described (Brown et al., 2018; Austin et al., 2019), only a few studies have shown persistent long-term cognitive dysfunction (Saczynski et al., 2012; Inouye et al., 2016). Saczynski et al. demonstrated an association between postoperative delirium and significantly lower postoperative cognition at both 1 month and 12 months following surgery (Saczynski et al., 2012). Inouye et al. demonstrated a significant decline in cognitive ability 36 months following postoperative delirium in a large cohort of 560 elective surgery patients without dementia at baseline (Inouye et al., 2016). Maclullich et al. reviewed the literature from 2003 to 2008 for long term cognitive decline in patients with acute in-hospital delirium. They concluded that stress responses, chronic neurodegeneration triggered by inflammation and infarct in critical brain areas can precipitate delirium acutely and long-term changes in cognition (MacLullich et al., 2009). Most of the studies reviewed diagnosed delirium using CAM and cognition using the mini mental state examination (MMSE). Considering that MoCA has higher sensitivity for diagnosing mild cognitive impairment and NCD in elderly patients, we chose to use the MoCA scoring in our study (Ciesielska et al., 2016; Siqueira et al., 2019).. Similar to our study, postoperative long term cognition using T-MoCA in non-emergent surgery was studied by Austin et al., concluding no difference between delirious and non-delirious patients at 90 days (Austin et al., 2019). In addition, while delirium is described as an independent risk factor for decline in functional ability, a significant decline has typically only been demonstrated at 1 month, but not at 12 months (Rudolph et al., 2010). Furthermore, these studies had larger samples and higher long-term retention rates than our study.

Our study has many important strengths. We conducted a rigorous randomized controlled trial with a factorial design and used state of the art delirium assessments to measure the primary outcome. With little missing data (<6% during the hospital stay) we were able to robustly quantify the effect of IV acetaminophen vs placebo combined with propofol or dexmedetomidine on postoperative delirium among older patients following cardiac surgery. We demonstrated a strong positive effect for prevention of delirium in the original study. For this follow-up study, we used a standardized measure (T-MoCA) to track cognitive and functional outcomes over 1-year by trained assessors, who were carefully blinded to the treatment group assignment.

However, several limitations are worthy of comment. First, the sample size is relatively small and was not explicitly powered to examine longer term cognitive or functional decline. The relatively small sample size and lack of power leads to an increased risk of a Type II error (Button et al., 2013). Moreover, a 1-year timeframe may have been too short to detect a significant long-term trajectory of cognitive decline. Additionally, there were losses to follow-up raising the possibility of informative censoring bias, such that patients with worse status failed to participate in the follow-up interviews, which may have affected our ability to elucidate the true relationship. It is also possible that additional measurements at three and/or 6 months would have enhanced retention of patients in the study and may have enabled us to better characterize cognitive changes over time. It is important to note that this was a single-site study, and the population enrolled were white and predominantly males, potentially limiting the generalizability. Finally, the interview used to collect data on the IADL and ADL status of patients during follow-up was adapted and did not include the multiple response categories typically used in the Lawton-Brody IADL test, which may have limited our ability to detect more subtle changes over time.

In the present analyses no statistically, significant improvement was demonstrated in the cognitive, depressive or functional outcomes at one and 12 months with the use of acetaminophen when compared to placebo. Given the limited sample size and participant retention in the study, further investigation is warranted. It will be important to further investigate both the long-term relationship between delirium with cognitive, emotional, and physical function, and also the long-term cognitive effects of acetaminophen in combination with dexmedetomidine or propofol following cardiac surgery. An interesting area of future research is to understand the molecular target of acetaminophen for delirium prevention. This would potentially provide a better understanding of acetaminophen’s ability to impact cognition and function over the long term. It would also be interesting to investigate other pharmacological and non-pharmacological interventions in reducing long term functional decline after delirium. Long-term outcome studies in vulnerable populations are challenging, and our study highlights the need for robust efforts to meet these challenges.

## Data Availability

The raw data supporting the conclusion of this article will be made available by the authors, without undue reservation.
